# Surgery

**Published:** 1909-03

**Authors:** Ernest W. Hey Groves


### Progress of the Medical Sciences.

### SURGERY.

A great deal of attention has been paid lately to the **surgery of the large intestine,** and the boldness with which the gut has been short-circuited or removed has directed much interest to the anatomy, physiology, and surgery of this part.

*Ileo-cæcal valve.*—Many recent observations point to the great importance of this structure. What is its normal function, how does it act, and how is it related to various pathological processes, are all questions which so far are not fully answered. That it is not the double-lipped slit, so long made familiar to us by diagrams and dried specimens, has been shown not only by hardening it *in situ* by formalin injections, but also by Keith's[1] observations on the part immediately after death. He found that by pouring hot wax into the ileum the valve closed by a definite sphincteric contraction of the circular muscle-fibres which surround the end of the small bowel and are contained in the lips of the valve, and there is a great probability that the opening and closure of the valve is regulated by conditions which obtain either in the cæcum or in the ileum. Elliott[2] has

[1] *J. Anat. and Physiol.*, 1904, xxxviii, p. viii.

[2] Elliott, *J. Physiol.*, 1904, xxxi, 157.

shown that the valve is held closed by a tonic contraction, and that active contractions of the lower ileum are accompanied by dilatation of the ileo-cæcal sphincter. When the splanchnic nerves are stimulated, the intestinal movements are inhibited, but the valve contracts more firmly. There can be no doubt that the junction between small and large bowels is more of the nature of a muscular sphincter than a mechanical valve, and this opens up interesting problems as to the rôle played by this structure in intestinal obstruction.

*The movements of the colon and its contents.*—For a long time it has been known that fluids may sometimes traverse the intestinal canal in a reverse direction to the normal, quite apart from intestinal obstruction. This was exemplified, *e.g.*, by Weber's[3] patient—an hysterical woman who vomited a coloured enema within ten minutes of its administration. Then in 1894 Grützner[4] showed that insoluble particles, such as chopped hair, suspended in normal saline and injected into the rectum, could be washed out of the stomach within six hours. But quite apart from this reverse fluid stream, it has been shown that in a part of the colon there exists a reverse peristalsis, which must exercise a very notable influence over many of the phenomena of colic disease. This was noticed first by Jacobi[5] accidentally when he was operating upon the abdomen of a dog. It was systematically investigated by Cannon[6] in America in cats, by means of feeding with bismuth mixed with porridge, which food throws a shadow by the X-rays on the fluorescent screen. The contractions occur in groups about every fifteen minutes, each contraction group lasting five minutes, and consisting of anti-peristaltic waves occurring four or five times each minute. Elliott and Barclay-Smith[7] have abundantly confirmed these observations on a great variety of animals, by actually watching the intestines after the abdomen has been opened. The anti-peristalsis begins usually towards the middle of the transverse colon, and travels back to the cæcum. In the majority of animals under an anæsthetic the movements are only seen after the spinal cord has been destroyed. If this abolition of the movements by an anæsthetic obtains in man, it accounts for the fact that they have not been observed during operations. But Hertz,[8] Mummery,[9] and Groves and Walker Hall[10] have made observations which conclusively show that this marked wave of anti-peristalsis is the chief and predominant movement of the colon

[3] Weber, *St. Barth. Hosp. Rep.*, xxix, 34.
[4] Grützner, *Deutsche med. Wchnschr.*, 1894, No. 48, p. 897.
[5] *Arch. f. exp. Path. u. Pharm.*, 1890. xxvii, 147.
[6] *Am. J. Physiol.*, vi, 253. [7] *J. Physiol.*, 1904, xxxi, 272.
[8] *Brit. M. J.*, 1908, i, 191.
[9] *Proc. Roy. Soc. Med., Surg. Sect.*, March, 1909. [10] *Ibid.*

in its proximal part in the human subject. And it has been pointed out by the last-named author that this anti-peristalsis will account for the symptoms of distension and pain which occur in the partially excluded colon, and which often necessitate the removal of this part of the bowel after ileo-sigmoidostomy. That is to say, if the ileum is cut in two near its end, and one end joined to the pelvic colon, the ascending colon and cæcum do actually in many cases become blown up by the movement of anti-peristalsis forcing fluid and gas into the blind pouch.

*The lymphatics of the colon.*—The course and arrangement of lymph vessels and glands have been thoroughly worked out by Jamieson and Dobson.[11] The general arrangement corresponds very closely with that of the blood-vessels, and can, therefore, be divided into three sets connected with (1) the cæcum and ascending colon, (2) the transverse colon, and (3) the descending, iliac and pelvic colon. The first set, which accompanies the ileo-colic artery, are divisible into two groups, an upper which is adjacent to the duodenum, and a lower near the ileo-cæcal junction. Sometimes the lymph vessels run direct from the cæcum to the glands of the upper group without joining the lower, and this fact makes it desirable to remove the whole series right up to the root of the mesentery when excising any malignant growth. The lymph vessels of the terminal six inches of the ileum join freely with those of the cæcum and appendix. In addition to the glands along the ileo-colic artery, there is an important series of glands closely adjacent to the large intestine, and called the para-colic set. These are specially numerous along the inner border of the ascending colon, but become fewer on the transverse, and fewer still on the descending and pelvic colon. The lymphatics of the transverse colon are contained in the transverse meso-colon, and those of the descending, iliac and pelvic colon lie with the inferior mesenteric vessels behind the parietal peritoneum. Thus each part of the large intestine is associated with a lymphatic area which is of a fan shape, the apex of the fan being situated in the middle of the transverse meso-colon at the root of the mesentery.

*Excision of malignant growths.*—The principles which have so long regulated the extent of radical operations for breast cancer have been applied now to cancer of the colon. Jamieson and Dobson[12] and Bird[13] have each successfully removed the whole of the cæcum, ascending colon, with parts of the transverse colon and ileum, for growths of the right colon. And together with this wide bowel removal the whole of the fan-shaped piece of peritoneum with its lymphatics has been excised up to the root of the mesentery. Schwijzer has applied the same principle to the transverse colon, the meso-colon being so thoroughly removed

[11] *Lancet*, 1907, i, 1137.

[12] *Lancet*, 1908, i, 149. [13] Bird, *Lancet*, 1906, i, 440.

that the pancreas was laid bare. His patient was alive and well two years after the operation. But in addition to these two factors necessary for permanent success, *i.e.* extensive removal of bowel above and below the growth and removal of the associated lymph area, there is a third which it is becoming increasingly clear is of the utmost importance, as without it the others can often not be carried out. This is the performance of a preliminary colotomy, or better still an ileo-sigmoidostomy, before the excision of the growth is attempted. This is not merely to relieve the distension of the bowel, it also places the patient in a far better position to undergo the radical operation, and this latter can be done quite freely without the fear of not being able to bring the ends of the gut together. The comparison of the results of operations performed in one or two stages is very much in favour of the latter, as far as the immediate results are concerned, and it is only reasonable to suppose that the ultimate results will be still more strikingly in favour of the operation *à deux temps*. It may be taken for granted that every surgeon would naturally prefer to do the whole operation at one sitting, producing a more brilliant result; but, nevertheless, even those who admit this feeling have to confess that the two-stage operation gives much better results. Bozelius, for example, gives twenty-eight cases at Breslau treated by secondary excision with only four deaths, whereas cases treated by primary resection had a mortality of 50 per cent. Putting together the results in cases described by Morton,[14] Bozelius,[15] Kummel,[16] Hochenegg[17] and Schloffer,[18] there were twelve cases of primary resection with five deaths, a mortality of 42 per cent., and fifty-seven cases of secondary resection with five deaths, a mortality of 9 per cent. And when we consider that the cases chosen for primary resection are those presenting the most favourable conditions, this comparison is still more striking.

*Functional diseases of the colon.* Under this term may be grouped all those cases where constipation is the main symptom, but where there is no primary organic disease. It includes idiopathic dilatation of the colon, primary constipation of colic origin and some forms of membranous colitis. Between the two former it is impossible to draw any hard-and-fast line. At the one extreme are those cases in children in which in earliest infancy the abdomen is enormously distended by huge coils of colon and which are clearly congenital. At the other are cases of adults in whom constipation becomes more and more marked, an[illegible] in whom the colon is found to be variously dilated, kinked a[illegible] distorted. Lowenstein[19] has collected over 150 cases of

[illegible]rit. *M. J.*, 1904, ii, 1149. [15] *Centralbl. f. d. ges. Therap.*, 1908, xxvi

[16] [17] [18] Quoted by Bozelius, *loc. cit.*

[19] *Centralbl. f. allge. Path.*, 1907, 929.

idiopathic dilatation from the literature, and he concludes that in some cases the disease is certainly congenital, but in others it is as certainly acquired. There are several points in which well-marked congenital cases differ from cases of acquired constipation with secondary dilatation. In the former the male sex greatly predominates, being in infants in the proportion of 7.1, and in adolescence 3.1. The growth and development of the chest is much stunted by the pressure of the abdominal contents during the time of growth, the abdomen is greatly distended and presents marked visible peristalsis, the general nutrition suffers early, and the end is almost always fatal. In the latter, on the other hand, the sex evidence is reversed, women being about six times more frequently affected than men, the chest is normal, the abdomen is but little distended, and active peristalsis is not seen, the general nutrition is maintained for many years, the subjects often being actually obese. In only a very few of these cases of primary acquired constipation in adults does a time arrive when the abdomen becomes distended and flesh and strength fail. As regards the treatment of all these cases a great deal has lately been done and written of which it will be possible here to give only the barest outline. Arbuthnot Lane[20] for years past has been advocating the surgical treatment by partial exclusion of the colon, and lately he has gone further and advised that the colon be in its greater part removed. It is very unfortunate that he has included in his series many cases of gastric ulcer and peritoneal adhesions following appendicitis—as these are clearly quite different from cases of simple chronic constipation. The mortality of 23 per cent. has shown that these heroic measures are only justifiable in the most extreme cases. Ito and Goyesima[21] and Tuffier[22] have also short circuited or excised the colon for conditions of dilatation, and their mortality is about 25 per cent.

Hertz[8] has shown by the X-rays, after bismuth feeding, that in many cases of chronic constipation the stasis occurs only in the pelvic colon, and that therefore in these cases to excise the upper portion of the large intestine would be to remove the only part that is working properly. One criticism may be urged against this, however, viz. that if the proximal part of the colon was excluded, the fæces would reach the pelvic colon in a liquid condition, with which it could deal much better than with solid masses. But it is certainly a point of great importance that the X-rays should be used systematically in these cases, in order to locate the position of stasis. Groves and Walker Ha[illegible][10] have shown that the metabolism was unaffected by exclusio[illegible] of

[20] *Brit. M. J.*, 1908, i, 126.

[21] *Deutsche Ztschr. f. Chir.*, 1907, lxx.

[22] *Bull. et Mem. de la Soc. de Chir. de Paris*, 1907.

[10] *Loc. cit.*

the greater part of the colon or by its removal in two cases, and the former has brought forward evidence from those cases which demonstrate that in the partially-excluded colon anti-peristalsis occurs, which often so distends the bowel as to cause great pain and toxæmia from the stagnation of its contents. Mansell Moullin[23] points out that simple lateral anastomosis between the ileum and pelvic colon has given perfectly satisfactory results in several cases of dilated colon under his care, and that in none of these has he had occasion to excise the colon subsequently, as happens so often when the ileum has been completely divided.

*The surgical treatment of colitis.*—Previous to 1895 the colon had sometimes been opened on the left side to drain conditions of ulcerative colitis (Hahn,[24] Robson,[25] Robinson,[26] Olmstead[27]). Then Keith,[28] and a little later Golding Bird,[29] practised a right-sided colostomy, which serves to divert the fæcal stream from practically the whole colon. The results from this latter operation have been almost uniformly good; but it necessitates the artificial anus being kept open for at least six months, and the fluid discharge from this is a source of great discomfort. Gibson,[30] in 1900, introduced a valvular cæcostomy,

[30] Gibson, *Boston M. and S. J.*, 1902, cxlvii, 341.

in which the cæcum was opened in the same way as in Kader's gastrostomy. But after a brief but successful career this operation was succeeded in 1902 by Weir's method of draining the large intestine through the stump of the appendix, and surgical literature has lately been full of the praises of this operation.

*Appendicostomy.*—Ever since its introduction this operation has been employed for various conditions of colitis—mucous, membranous, ulcerative, dysenteric, and tuberculous—and all who have had experience of it speak in its favour. Weir,[31] Meyer,[32] Lilienthal,[33] Hutchinson,[34] Bennett,[35] Murray,[36] Dawbarn,[37] Moynihan,[38] Bishop,[39] Armour,[40] Lett,[41] Sumner[42] Waterhouse[43] and Keetley,[44] have published cases illustrating its method, and the last-named has carefully described its technique.

[23] *Lancet*, 1909, i, 156.

[24] Hahn, *Arch. f. klin. Chir.*, 1893, xxix, 395.

[25] *Lancet*, 1893, i, 1164. [26] *Ibid.*, *Tr. Clin. Soc. Lond.*, 1893, xxvi, 213.

[27] *Brit. M. J.*, 1906, ii, 1277. [28] *Lancet*, 1896.

[29] *Tr. Clin. Soc. Lond.*, 1896, xxix, 45; 1899, xxxii, 183; 1902, xxxv, 164; *Brit. M. J.*, 1899, i, 1,217; 1895, ii, 1559.

[31] *Med. Rec.*, 1902, lxii, 201. [32] *Med. News*, 1905, 405.

[33] Quoted by Olmsted, *loc. cit.* [34] *Brit. M. J.*, 1905, i, 1039.

[35] *Lancet*, 1906, i, 419. [36] *Ann. Surg.*, 1901, xxxiii, 579.

[38] [39] [40] Quoted by Lett. [41] *Treatment*, 1905, ix, 881.

[42] Quoted by Lett. [43] *Proc. Roy. Soc. Med.*, March, 1909.

[44] *Ibid.*

In the less severe grades of colitis it may act as an absolute cure, but in the more severe chronic forms of ulceration its results are not so lasting; and for such cases Pauchet and Prieur[45] have adopted the operation of ileo-sigmoidostomy with most excellent results. Keetley[44] has gone much further than other authors in extolling the possibilities of the operation, so that he even suggests that as much of the appendix should be preserved as possible in acute appendicitis, and fixed in the parietal wound for future use, in case the need for appendicostomy should arise. The possibility of using the stump of the appendix for saline infusion in cases of diffuse peritonitis is a very important point, and one the value of which is shown by cases described by Billington. And lastly, this operation has been employed in cases of obstinate constipation, both to wash out the large intestine and for the introduction of purgatives directly into the gut. So far the operation, performed in uncomplicated cases, has no mortality, and the results from it are so good that it will probably take its place as a most valuable addition to our means of treating various disordered conditions of the colon; and if in any case it is unsuccessful, it can be followed by the more severe measures of ileo-sigmoidostomy or excision.

*Pericolitis and diverticulitis.*—No account of recent work on the surgery of the large intestine would be complete without reference to this subject. But it has recently been so fully dealt with by Telling[46] in the pages of the *Lancet*, that it will only be necessary here to refer to some of the main points. The diverticula are almost always acquired, and are rarely found except in elderly people. Each diverticulum varies in size from that of a tiny pouch barely visible to the naked eye to that of a cherry. They are generally multiple, and may be present to the number of several hundreds. They are most numerous in the pelvic colon, and diminish in frequency in the higher parts of the bowel, so that they are rare in either the transverse or ascending colon. Each diverticulum consists of a pouching of the mucous membrane outwards through the thinned muscular coat, and a covering of peritoneum, except when the diverticula occur in the non-serous surface of the gut, when the latter is absent. In the great majority of cases they project into the interior of the appendical epiploicæ. When fully formed, such a diverticulum presents conditions, the pathological possibilities of which are exactly similar to those of the appendix vermiformis. These may be summarised as follows: (1) General peritoneal infection without perforation; (2) acute gangrenous diverticulitis; (3) local plastic peritonitis; (4) general perforative peritonitis; (5) adhesions to other viscera which may cause acute intestinal obstruction; (6) local abscess, which resembles

[45] *Arch. Prov. de Chir.*, No. 11, 1905.

[46] *Lancet*, 1908, i, 843, 928.

a left-sided appendicitis; (7) chronic sigmoid mesenteritis which ties the colon down to the pelvic wall in a mass of adhesions; (8) chronic sigmoiditis, forming a hard, annular fibrous mass resembling carcinoma, and producing, like the latter, chronic intestinal obstruction; (9) carcinoma may actually develop at the site of a chronic sigmoiditis. It is probable that many cases which have been described as cancer of the sigmoid are really examples of chronic inflammation round diverticula, and, indeed, this has been demonstrated in some museum specimens.

ERNEST W. HEY GROVES.

